# The Effect of Daily Iron Supplementation with 60 mg Ferrous Sulfate for 12 Weeks on Non-Transferrin Bound Iron Concentrations in Women with a High Prevalence of Hemoglobinopathies

**DOI:** 10.3390/jcm8020180

**Published:** 2019-02-03

**Authors:** Shannon L. Steele, Hou Kroeun, Crystal D. Karakochuk

**Affiliations:** 1 Food, Nutrition and Health, The University of British Columbia, Vancouver, BC V6T 1Z4, Canada; shannon.steele@ubc.ca; 2 British Columbia Children’s Hospital Research Institute, Vancouver, BC V5Z 4H4, Canada; 3 Helen Keller International, Phnom Penh, Cambodia; hkroeun@hki.org

**Keywords:** anemia, hemoglobinopathy, iron, non-transferrin bound iron, supplementation, toxicity, transferrin saturation

## Abstract

There is a lack of evidence for the safety of untargeted daily iron supplementation in women, especially in countries such as Cambodia, where both anemia and hemoglobinopathies are common. Our aim was to assess serum non-transferrin bound iron (NTBI), a toxic biochemical that accumulates in blood when too much iron is absorbed, in Cambodian women who received daily iron supplements in accordance with the 2016 global World Health Organization (WHO) guidelines. We used fasting venous blood samples that were collected in a 2015 supplementation trial among predominantly anemic Cambodian women (18–45 years). Serum NTBI was measured with use of the FeROS™ eLPI assay (Aferrix Ltd., Tel-Aviv, Israel) in randomly selected sub-groups of women who received 60 mg daily elemental iron as ferrous sulfate (*n* = 50) or a placebo (*n* = 50) for 12 weeks. Overall, *n* = 17/100 (17%) of women had an elevated serum NTBI concentration (≥0.1 μmol/L) at 12 weeks; *n* = 9 in the Fe group and *n* = 8 in the placebo group. Elevated serum NTBI concentration was not associated with age, iron supplementation, transferrin saturation or severe hemoglobinopathies (*p* > 0.05). In this population of women with a high prevalence of hemoglobinopathies, we found that daily iron supplementation was not associated with elevated serum NTBI concentrations at 12 weeks, as compared to placebo.

## 1. Introduction

It is often assumed that approximately half of the anemia burden in low-income countries is due to iron deficiency, which has been the driving force for the World Health Organization (WHO) to establish recommendations for untargeted iron supplementation among non-pregnant women. These recommendations include the 2016 WHO guideline recommending daily iron and folic acid (60 mg elemental iron) for three consecutive months of the year for all adolescent girls and menstruating women in areas of anemia prevalence ≥40% [1].

The benefits of iron supplementation in iron-deplete women are well established. However, most studies investigating the efficacy of untargeted iron supplementation fail to assess the potential harms beyond gastrointestinal side effects. However, it should be acknowledged that iron is a catalyst of oxidative and inflammatory reactions, and excess iron can cause intestinal injury and oxidative stress [2]. Iron toxicity has also been associated with diabetes, certain cancers, and neuropathies. Thus, there is a justified need to evaluate if there is potential harm associated with untargeted iron supplementation.

Furthermore, several recent observational surveys in a number of low-resource countries have shown an unexpectedly low prevalence of iron deficiency in non-pregnant women (<8% based on inflammation-adjusted ferritin) [3]. If iron deficiency is not a major cause of anemia in these populations, then at best untargeted iron supplementation is a waste of resources, and at worst, it could cause harm, especially in iron-replete women or those with hemoglobinopathies who are already at risk of altered iron homeostasis [4]. Genetic hemoglobinopathies are autosomal recessive disorders that cause a reduced production, or a defective hemoglobin structure (resulting in anemia) [5,6]. Hemoglobinopathies, such as α-thalassemia or hemoglobin E variants, are prevalent in Cambodia (~60%) [7], and other variants are common in other regions in the world (e.g., sickle cell disease in parts of Africa) [8,9]. In some forms of hemoglobinopathies, ineffective erythropoiesis and down-regulation of hepcidin expression can contribute to increased iron absorption, irrespective of iron status [4,10]. Because of this, individuals with hemoglobinopathies are at an increased risk of iron overload and toxicity [4].

Iron in circulation is normally bound to transferrin, ferritin, or hemoglobin [11]. However, when the rate of iron influx into plasma exceeds the rate of iron acquisition by transferrin, free unbound iron is produced, called non-transferrin bound iron (NTBI) [12,13]. This is a circulating free form of iron that is redox-active and able to catalyze the formation of reactive oxygen species [14]. Circulating NTBI is potentially toxic due to its propensity to induce oxidative damage to the plasma membrane and intracellular organelles [14]. The production of NTBI appears to be strongly influenced by the rate and amount of iron absorbed [15]. With just one 60-mg bolus of ferrous sulfate, Brittenham et al. [15] found that NTBI concentrations remained elevated in healthy women at 8 h post ingestion of iron (60 mg elemental iron), which suggests clearance of iron at this time was still incomplete. No published studies to date have measured NTBI concentrations after 12 weeks of daily oral iron supplementation at 60 mg (dose and duration as per the WHO policy) in healthy individuals or those with hemoglobinopathies.

The aim of the current study was to measure serum NTBI concentrations in Cambodian women with a high prevalence of hemoglobinopathies who were supplemented with daily oral iron (in accordance with the recent 2016 WHO policy), or a placebo, for 12 weeks.

## 2. Experimental Section

### 2.1. Study Design and Population

Fasting venous blood samples that were collected as part of a supplementation trial in 2015 among 401 predominantly anemic Cambodian women were used. The full trial has been published elsewhere [16]. Inclusion criteria included healthy, non-pregnant women aged 18-45 years who had a hemoglobin concentration <117 g/L at screening based on a non-fasting capillary blood sample (Hemocue 301, Hemocue AB, Ängelholm, Sweden). Exclusion criteria included women who were taking medication or food supplements. Women were recruited to the trial from 26 villages in Kampong Chhnang province in Cambodia.

For the current study, we randomly selected *n* = 50 women who received 12 weeks of daily oral iron as 60 mg elemental iron as ferrous sulfate (the Fe group), and *n* = 50 women from the placebo group. Women were advised to consume the capsules with food and adequate fluids. We hypothesized that women who received daily oral iron (60 mg) for 12 weeks would have a higher mean serum NTBI concentration, as compared to women who received a placebo.

### 2.2. Ethical Approval

Ethical approval for the supplementation trial was obtained from the University of British Columbia Clinical Research Ethics Board in Canada for the iron trial (H15-00933) and the current study (H17-02650), and the National Ethics Committee for Health Research in Cambodia (110-NECHR). The trial was registered at clinicaltrials.gov (NCT-02481375).

### 2.3. Blood Collection and Analysis

Fasting venous blood was collected at baseline and at 12 weeks in a 6 mL trace element-free tube for serum, a 6 mL evacuated EDTA tube for plasma, buffy coat, and RBCs, and a 2 mL EDTA tube for a complete blood count. Samples were stored at −80 °C until shipment or analysis. Genetic hemoglobinopathies, and concentrations of hemoglobin, ferritin, hepcidin, and other biomarkers of nutritional status and inflammation were measured and have been reported elsewhere [16].

In the current study, we measured serum NTBI with use of the FeROS™ eLPI assay kit (Aferrix Ltd., Tel-Aviv, Israel). The FeROS™ assay has been validated in an international round robin for NTBI [17]. Non-transferrin bound iron is measured on a 96-well plate as labile plasma iron (LPI) with use of a fluorescence plate reader and reported as µmol/L. Serum iron (µmol/L) and total iron-binding capacity (TIBC, µmol/L) were measured using inductively coupled plasma mass spectrometry (Agilent 7500ce, Agilent Technologies, Tokyo, Japan) at the University of Otago in New Zealand. Transferrin saturation (TSAT, %) was calculated as the value of serum iron divided by TIBC and multiplied by 100.

### 2.4. Statistical Analyses

Descriptive statistics (mean ± SD, median (IQR), and proportions) were used to describe the characteristics of the population. An elevated serum NTBI was defined as a serum NTBI concentration ≥0.1 µmol/L. *T*-tests (for parametric) and Wilcoxon rank-sum tests (for non-parametric) were used to compare concentrations of nutritional and hematological biomarkers between the Fe and placebo groups. Chi-square tests or Fischer’s exact tests (when expected cell frequencies in the 2 × 2 table were <5) were used to compare prevalence rates (e.g., prevalence of genetic hemoglobin disorders) across the Fe and placebo groups. Ferritin was corrected for sub-clinical inflammation using inflammation biomarkers (α-1 acid glycoprotein and C-reactive protein) [18]. A mixed-effects logistic regression model was used to determine the factors (age, transferrin saturation, presence or absence of a genetic hemoglobin EE disorder, and iron or placebo intervention) associated with an elevated serum NTBI concentration at 12 weeks (binary outcome), adjusting for random effects (villages). Stata 15 (College Station, TX, USA) was used.

## 3. Results

### 3.1. Baseline Characteristics

Baseline characteristics, biomarkers of nutritional and inflammation status are presented in Table 1. No statistical differences were observed between Fe and placebo groups. Of the *n* = 100 women, a total of 82% of the women were iron-replete (*n* = 82/100, ferritin >15 µg/L) and 67% (*n* = 67/100) had some form of a hemoglobinopathy. Of those *n* = 67 with a hemoglobinopathy, 9% (*n* = 6/67) had a hemoglobin EE homozygous disorder, a severe disorder that has been shown to be associated with altered iron metabolism. 

### 3.2. Indicators of Hematological and Nutritional Status at Baseline and 12 Weeks

We assessed the indicators of hematological and nutritional status among women at baseline and at 12 weeks, and the proportion of women with elevated serum NTBI concentrations among the *n* = 100 women at 12 weeks (Table 2). Overall, *n* = 39/100 (39%) of women had anemia (hemoglobin <120 g/L) and *n* = 18/100 (18%) had iron deficiency (inflammation-adjusted ferritin <15 µg/L) at baseline.

### 3.3. Proportion of Women with an Elevated Serum NTBI Concentrations at 12 Weeks

Overall, 17% (*n* = 17/100) of women had an elevated serum NTBI concentration at 12 weeks, as defined as a serum NTBI concentration ≥0.1 µmol/L: *n* = 9/50 in the Fe group and *n* = 8/50 in the placebo group. Nearly all women (94%; *n* = 16/17) had a serum NTBI value equivalent to 0.1 µmol/L at 12 weeks, except for *n* = 1 woman who had a concentration of 0.7 µmol/L in the placebo group. 

Transferrin saturation (%) was not independently associated with an elevated serum NTBI concentration at 12 weeks (*p* = 0.17; Wilcoxon rank-sum test for nonparametric data).

The proportion of women with an elevated serum NTBI concentration at 12 weeks was not significantly different among those with any type of a genetic hemoglobinopathy (*n* = 9/67; 13%) than those with no hemoglobinopathies (*n* = 8/33; 24%) (*p* = 0.18; Pearson’s chi-square test).

### 3.4. Factors Associated with Elevated Serum NTBI Concentrations at 12 Weeks

We used a mixed-effects logistic regression model to determine the factors associated with an elevated serum NTBI concentration, as defined as a serum NTBI concentration ≥0.1 µmol/L. None of the factors we assessed were significantly associated with elevated serum NTBI concentrations at 12 weeks (Table 3).

## 4. Discussion

In this study population of predominantly anemic women with a high prevalence of genetic hemoglobinopathies, we found that 12 weeks of iron supplementation with 60 mg elemental iron as ferrous sulfate was not associated with elevated serum NTBI concentrations, as compared to a placebo.

Only *n* = 17/100 women had an elevated serum NTBI concentrations at 12 weeks, and this did not appear to be due to iron supplementation (*n* = 9 women in the Fe and *n* = 8 in the placebo group). We acknowledge some important considerations that may have hindered our ability to observe a significantly higher mean NTBI concentration in women receiving the iron supplements. First, we collected fasting blood samples from women in the early morning; thus, it was most likely that they consumed their last iron supplement during the evening meal of the previous day, at least 12+ h before the time of fasting blood collection. Second, we advised women to consume the supplements with food and adequate fluid, to mitigate the potential for adverse gastrointestinal side effects as a result of the high iron dose in the iron arm (60 mg elemental iron).

Brittenham et al. have demonstrated that serum NTBI accumulation is substantially reduced in healthy women consuming 60 mg of elemental iron when the iron supplement is consumed with food [15]. Further, the accumulation of NTBI appears to peak higher in healthy women receiving 60 mg elemental iron at ~2–4 h without food, as compared to women consuming iron with food [15]. Therefore, due to the timing of blood sample collection in our study and because we advised women to consume supplements with food, we may have mitigated the potential effect of iron on serum NTBI accumulation in the blood of women who consumed iron. If women in our study consumed the iron capsule with food (as instructed), we suspect that serum NTBI concentrations at 12 weeks would be lower as compared to women who consumed the iron capsule without food. A more accurate assessment would include the measurement of NTBI concentrations at ~2 h post ingestion (considered the ‘peak’) and examination of the effects of iron on NTBI concentrations with and without food consumption.

The type of food consumed with the iron supplement may also affect the levels of NTBI accumulation. For example, phytates, a component of plant-foods that bind to and inhibit iron absorption, are found in many staple foods in low-resource countries (cereals, legumes, seeds) [19]. Phytates have a high affinity for iron; thus, if individuals taking iron supplements are also consuming phytate-rich diets, iron bioavailability may be decreased [20], and this may result in reduced serum NTBI accumulation as compared to individuals taking iron supplements and consuming phytate-poor diets. In Cambodia, diets are generally considered to be low in phytate content, as white rice is the common staple food and is low in phytates. However, this may be a factor to consider in other populations with diets high in phytate or other dietary components that reduce iron bioavailability and absorption.

We did not see a significant association between serum transferrin saturation (%) and serum NTBI concentrations at 12 weeks (*p* = 0.17); which is contrary some of the published literature [21]. Transferrin saturation is a composite measure of serum iron and total iron binding capacity, and is a strong predictor of iron overload in clinical practice. However, other studies have shown the NTBI can accumulate in the absence of an elevated transferrin saturation, if the rate of iron influx into the blood exceeds the rate of iron acquisition by transferrin [12,13,15]. Also, we may have been underpowered to detect an association, given the relatively small number of women in our study with an elevated serum NTBI concentration (*n* = 17).

We acknowledge the limitation in our study that we did not measure baseline serum NTBI concentrations, which would have led to a more rigorous analysis at the end of our 12 weeks intervention.

We used ferrous sulfate as the form of iron supplement in our study; however, other forms of iron may have a different effect on serum NTBI concentrations. Currently, ferrous sulfate and ferrous fumarate are the most common forms of iron supplements used in low-resource countries due to their low cost and availability on the WHO List of Essential Medicines as an antianemia medicine [22]. However, other novel forms of supplemental oral iron, such as NaFeEDTA, have shown to be more bioavailable and associated with lower concentrations of NTBI accumulation post-ingestion, as compared to iron-bound salts [21,23]. Research is warranted to investigate the risk of NTBI accumulation from supplementation with other novel forms of iron (e.g., amino acid chelates) [24]. Lastly, more research is needed to ascertain the level at which concentrations of NTBI are associated with the highest risk of adverse outcomes in individuals.

The aim of this study was to determine if there was an increased risk of untargeted iron supplementation in Cambodian women with a high prevalence of hemoglobinopathies who received 12 weeks of daily oral iron (in accordance with the recent 2016 WHO policy), as compared to a placebo. We did not observe any significant risk as measured by an elevated serum NTBI concentration in this studied population. However, we do acknowledge that we did observe benefits: Women in the Fe group had significantly increased ferritin concentrations at 12 weeks, and iron deficiency prevalence in this group decreased from 16% to 2% after 12 weeks of supplementation. Ultimately, the rationale for blanket supplementation programs should be ascertained with data on both the associated risks and benefits of the intervention. We conclude that other biomarkers or tests that measure the potential risk of iron supplementation could be trialed (e.g., gut microbiome, DNA damage).

## Figures and Tables

**Table 1:** Baseline characteristics of enrolled non-pregnant Cambodian women (18–45 years). ^1^

	Fe	Placebo	*p* Value
Total *n* (%)	50 (50%)	50 (50%)	
Age, years, mean ± SD	30.4 ± 7.3	31.2 ± 8.6	0.62
Parity, *n* of children born, median (IQR)	1.5 (0, 3.0)	1.0 (0, 3.0)	0.73
Household size, *n*, mean ± SD	4.5 ± 1.6	4.6 ± 1.3	0.63
Hemoglobin concentration, g/L, median (IQR)	125 (117, 121)	121 (115, 127)	0.37
Ferritin concentration, ^2^ µg/L, median (IQR)	46.4 (18.9, 80.6)	38.3 (23.8, 58.6)	0.59
Transferrin saturation, %, median (IQR)	23.7 (14.6, 31.5)	21.2 (12.5, 27.0)	0.39
Prevalence of a hemoglobinopathy, ^3^ *n* (%)	36/50 (72%)	31/50 (62%)	0.29

^1^ Total *n* = 100 women. Fe, iron; IQR, interquartile range. ^2^ Ferritin was corrected for sub-clinical inflammation using inflammation biomarkers (α-1 acid glycoprotein and C-reactive protein). ^3^ Including genetic hemoglobin variants in heterozygous or homozygous form (hemoglobin E, Constant Spring, H, Bart or F), or α-thalassemia. T-tests (for parametric) and Wilcoxon rank-sum tests (for non-parametric) were used to compare concentrations of nutritional and hematological biomarkers. Chi-square tests or Fischer’s exact tests (when expected cell frequencies in the 2 × 2 table were <5) were used to compare prevalence rates (i.e., prevalence of a hemoglobinopathy) across the Fe and placebo groups.

**Table 2:** Indicators of hematological and nutritional status among women. ^1^

	Fe	Placebo	*p* Value
Total *n* (%)	50 (50%)	50 (50%)	
Anemia (hemoglobin <120 g/L), *n* (%)			
At baseline	20/50 (40%)	19/50 (38%)	0.84
At 12 weeks	14/50 (28%)	26/50 (52%)	0.01
Iron deficiency (ferritin <15 µg/L), ^2^ *n* (%)			
At baseline	8/50 (16%)	10/50 (20%)	0.60
At 12 weeks	1/50 (2%)	10/50 (20%)	0.01
Transferrin saturation, %, median (IQR)			
At baseline	23.7 (14.6, 31.5)	21.3 (12.5, 27.0)	0.39
At 12 weeks	26.6 (20.9, 32.2)	19.5 (11.0, 27.6)	0.001
Elevated serum NTBI (≥0.1 µmol/L), *n* (%)			
At 12 weeks ^3^	9/50 (18%)	8/50 (16%)	0.79

^1^ Total *n* = 100 women. Fe, iron; IQR, interquartile range; NTBI, non-transferrin bound iron. ^2^ Ferritin was corrected for sub-clinical inflammation using inflammation biomarkers (α-1 acid glycoprotein and C-reactive protein). ^3^ Serum NTBI concentrations were only measured at 12 weeks (not at baseline). Chi-square tests or Fischer’s exact tests (when expected cell frequencies in the 2 × 2 table were <5) were used to compare prevalence rates (e.g., prevalence of genetic hemoglobin disorders) across the Fe and placebo groups.

**Table 3:** Factors associated with elevated serum non-transferrin bound iron (NTBI) concentrations at 12 weeks ^1^.

Factor	OR (95% CI)	SE	*p* Value
Age, years	0.98 (0.91, 1.05)	0.036	0.55
Transferrin saturation, %	1.04 (0.99, 1.09)	0.024	0.12
Presence of a homozygous Hb EE disorder	0.95 (0.09, 9.58)	1.121	0.97
Iron supplementation (60 mg for 12 weeks)	0.92 (0.30, 2.85)	0.531	0.89
Constant	0.16 (0.01, 2.02)	0.207	0.16

^1^ A mixed-effects logistic regression model was used to determine which factors were associated with an elevated serum NTBI concentration at 12 weeks, defined as ≥0.1 µmol/L. CI, confidence ratio; Hb, hemoglobin; NTBI, non-transferrin bound iron; OR, odds ratio; SE, standard error.

## References

[1] (2016). Guideline: Daily Iron Supplementation in Adult Women and Adolescent Girls.

[2] Schümann K., Ettle T., Szegner B., Elsenhans B., Solomons N.W. (2007). On risks and benefits of iron supplementation recommendations for iron intake revisited. J. Trace Elem. Med. Biol..

[3] Karakochuk C.D. (2016). Iron supplementation in predominantly iron-replete populations: Is there an emerging concern?. Sight Life Mag..

[4] Zimmermann M.B., Fucharoen S., Winichagoon P., Sirankapracha P., Zeder C., Gowachirapant S., Judprasong K., Tanno T., Miller J.L., Hurrell R.F. (2008). Iron metabolism in heterozygotes for hemoglobin E (HbE), alpha-thalassemia 1, or beta-thalassemia and in compound heterozygotes for HbE/beta-thalassemia. Am. J. Clin. Nutr..

[5] Bain B.J. (2006). Haemoglobinopathy Diagnosis.

[6] Greer J.P., Foerster J., Rodgers G.M., Paraskevas F. (2009). Wintrobe’s Clinical Hematology.

[7] Karakochuk C.D., Whitfield K.C., Barr S.I., Lamers Y., Devlin A.M., Vercauteren S.M., Kroeun H., Talukder A., McLean J., Green T.J. (2015). Genetic hemoglobin disorders rather than iron deficiency are a major predictor of hemoglobin concentration in women of reproductive age in rural Prey Veng, Cambodia. J. Nutr..

[8] Weatherall D.J., Clegg J.B. (2001). Inherited haemoglobin disorders: An increasing global health problem. Public Health Rev..

[9] Piel F.B., Patil A.P., Howes R.E., Nyangiri O.A., Gething P.W., Williams T.N., Weatherall D.J., Hay S.I. (2010). Global distribution of the sickle cell gene and geographical confirmation of the malaria hypothesis. Nat. Commun..

[10] Kattamis A., Papassotiriou I., Palaiologou D., Apostolakou F., Galani A., Ladis V., Sakellaropoulos N., Papanikolaou G. (2006). The effects of erythropoetic activity and iron burden on hepcidin expression in patients with thalassemia major. Haematologica.

[11] Frazer D.M., Anderson G.J. (2014). The regulation of iron transport. BioFactors.

[12] Hutchinson C., Liu D.Y., Hider R.C., Powell J.J., Geissler C.A. (2004). Oral ferrous sulphate leads to a marked increase in pro-oxidant nontransferrin-bound iron. Eur. J. Clin. Investig..

[13] Hod E.A., Brittenham G.M., Billote G.B., Francis R.O., Ginzburg Y.Z., Hendrickson J.E., Jhang J., Schwartz J., Sharma S., Sheth S. (2011). Transfusion of human volunteers with older, stored red blood cells produces extravascular hemolysis and circulating non-transferrin-bound iron. Blood.

[14] Brissot P., Ropert M., Le Lan C., Loréal O. (2012). Non-transferrin bound iron: A key role in iron overload and iron toxicity. Biochim. Biophys. Acta.

[15] Brittenham G.M., Andersson M., Egli I., Foman J.T., Zeder C., Westerman M.E., Hurrell R.F. (2014). Circulating non-transferrin-bound iron after oral administration of supplemental and fortification doses of iron to healthy women: A randomized study. Am. J. Clin. Nutr..

[16] Karakochuk C.D., Barker M.K., Whitfield K.C., Barr S.I., Vercauteren S.M., Devlin A.M., Hutcheon J.A., Houghton L.A., Prak S., Hou K. (2017). The effect of oral iron with or without multiple micronutrients on hemoglobin concentration and hemoglobin response among non-pregnant Cambodian women of reproductive age: A 2 × 2 double-blind randomized controlled supplementation trial. Am. J. Clin. Nutr..

[17] Jacobs E.M.G., Hendriks J.C.M., Van Tits B.L.J.H., Evans P.J., Breuer W., Ding Y.L., Jansen E.H.J.M., Jauhiainen K., Sturm B., Porter J.B. (2005). Results of an international round robin for the quantification of serum non-transferrin-bound iron: Need for defining standardization and a clinically relevant isoform. Anal. Biochem..

[18] Thurnham D.I., McCabe L.D., Haldar S., Wieringa F.T., Northrop-Clewes C.A., McCabe G.P. (2010). Adjusting plasma ferritin concentrations to remove the effects of subclinical inflammation in the assessment of iron deficiency: A meta-analysis. Am. J. Clin. Nutr..

[19] Petry N., Egli I., Zeder C., Walczyk T., Hurrell R. (2010). Polyphenols and phytic acid contribute to the low iron bioavailability from common beans in young women. J. Nutr..

[20] Gibson R.S., Raboy V., King J.C. (2018). Implications of phytate in plant-based foods for iron and zinc bioavailability, setting dietary requirements, and formulating programs and policies. Nutr. Rev..

[21] Schümann K., Kroll S., Romero-Abal M.E., Georgiou N.A., Marx J.J.M., Weiss G., Solomons N.W. (2012). Impact of oral iron challenges on circulating non-transferrin-bound iron in healthy guatemalan males. Ann. Nutr. Metab..

[22] World Health Organization (2017). Model List of Essential Medicines.

[23] Schümann K., Solomons N.W., Orozco M., Romero-Abal M.E., Weiss G. (2013). Differences in circulating non-transferrin-bound iron after oral administration of ferrous sulfate, sodium iron EDTA, or iron polymaltose in women with marginal iron stores. Food Nutr. Bull..

[24] Milman N., Jønsson L., Dyre P., Pedersen P.L., Larsen L.G. (2014). Ferrous bisglycinate 25 mg iron is as effective as ferrous sulfate 50 mg iron in the prophylaxis of iron deficiency and anemia during pregnancy in a randomized trial. J. Perinat. Med..

